# Can the Human Resources Index (HRI) Be Used as a Process Feedback Measurement in a Structured Support Model for Systematic Work Environment Management?

**DOI:** 10.3390/ijerph18126509

**Published:** 2021-06-16

**Authors:** Fredrik Molin, Sofia Åström Paulsson, Therese Hellman, Magnus Svartengren

**Affiliations:** 1 IPF, the Institute for Organizational and Leadership Development at Uppsala University, 753 20 Uppsala, Sweden; 2 Department of Medical Sciences, Occupational and Environmental Medicine, Uppsala University, 752 37 Uppsala, Sweden; sofia.astrom_paulsson@medsci.uu.se (S.Å.P.); therese.hellman@medsci.uu.se (T.H.); magnus.svartengren@medsci.uu.se (M.S.)

**Keywords:** systematic work environment management, work environment, relational justice, short-term recovery, work environment-related production loss, health-related production loss

## Abstract

The aim of the study was to estimate the level of the human resources index (HRI) measure among Swedish municipal employees, and to investigate the association between human resources index (HRI) and relational justice, short-term recovery, work environment-related production loss, and health-related production loss. A cross-sectional design was used with one sample of municipal employees (*n* = 6402). The results showed a positive association (r = 0.31) between human resources index (HRI) and relational justice; a positive (r = 0.27) association between HRI and short-term recovery; a negative association between HRI and work environment-related production loss (r = −0.37); and a negative association between HRI and health-related production loss (r = −0.23). The findings implicate that HRI captures important aspects of the work environment such as productivity, relational justice, and short-term recovery. The HRI measure is part of a support model used in workplaces to systematically address work environment-related issues. Monitoring changes in the HRI measure, it is possible to determine whether the measures taken effect production loss, perceived leadership, and short-term recovery in a work group. The support model using HRI may thus be used to complement traditional work environment surveys conducted in Swedish organizations as obliged by legal provisions.

## 1. Introduction

Work environment surveys investigating the perceived work environment of the employees are becoming an established part of the systematic work environment management performed in Swedish organizations. Swedish provisions on systematic work environment management states that employers are required to examine risks in the work environment in a systematic manner, following an established cycle of risk assessment, measures, and follow up (AFS 2001:1) [1]. However, studies indicate that the provision is not implemented in all sectors in Sweden [2], and the integration of systematic work environment management within Swedish organizations remain weak [3]. Despite the growing expectation that work environment surveys will respond to legal requirements and provide ground for better decisions regarding work environment issues, many organizations do not implement the results from work environment surveys in their work environment management; moreover, there is considerable uncertainty over how best to use them. Negative factors and risks in the work environment are thus dutifully identified by Swedish organizations, but the steps concerning measures and evaluation of effects of the taken measures are absent.

Previous research has indicated several important factors influencing the work environment in organizations such as job demand, job control, perceived fairness in the organization, work environment-related productivity loss, and short-term recovery [4,5,6,7].

One of the most widely used models for explaining the impact of the psychosocial work environment on health is the demand control support model [4]. The model shows that the health of employees is negatively linked to high work demands and positively linked to experiences of control and social support in the workplace. Influence is thus an important work environment factor [8,9]. There is clear support for the link between work requirements, perceived control, and employee health [10,11]. A similar model to the demand control support model is proposed by Bakker and Demerouti [12], focusing on job demand and available resources at a workplace (JD-R).

Another important aspect of the social work environment is the perceived fairness in the organization, often referred to as organizational justice. The concept of organizational justice is divided into four dimensions: distributive justice, procedural justice, interpersonal justice, and informative justice [5,13,14]. In the present study, the focus is on interpersonal justice (also referred to as relational justice or interactional justice), which emphasizes the superior’s relationship to the employees, for example, how the senior manager handles the employees’ personal views and rights; and if the employees are treated impartially, truthfully, and with kindness. Relational justice is thus being treated fairly at the workplace and has been shown to be linked with health in the workplace [15]. In several extensive studies, Finnish researchers have examined the connection between organizational justice and several health-related parameters such as sick absence [16], long-term inflammatory markers [17], smoking [18], and alcohol consumption [19]. Relationships have been shown between mental illness and relational justice and procedural justice [5,16], and the degree to which employees are treated fairly in the workplace has been shown to be able to predict future ill health [16], self-rated health and burnout [20], and metabolic syndrome [21]. A relation between perceived leadership and cardiovascular disease is also found in previous research [22], and employees in companies with a high rated justice index also have a higher rated health and well-being [23,24]. Leadership, social climate, and commitment are also important factors contributing to employee well-being [25]. The model for organizational justice has conceptual connections to the above-mentioned demand control support model [5].

Recovery has been shown to be an important factor in stress prevention [7], and good recovery has been shown to be negatively correlated with emotional exhaustion [26]. The ability to sleep well and recover is affected by work-related stress of various kinds [27]; furthermore, an increased recovery paves the way for greater resilience to challenges in everyday life and reduced risk of mental illness and future production loss. Recovery is defined as the time needed for an individual to fully recover from stress and function normally on a prestress level [28]. Quality of sleep is an important part of recovery from stress and is made up of sleep duration and sleep continuity. Feeling refreshed when waking up is positively associated with objective sleep quality [29]. Impaired sleep quality is linked to health outcomes such as sick absence [30], diabetes, and cardiovascular disease [31]. Other studies also indicate a negative association between sleep quality and stress [32,33,34]. Linton et al. [35] found that psychosocial variables at work such as organizational justice, social support, and control were linked to less sleep disturbances among employees.

The aspects of production loss refer to the fact that individuals may be present at work but have reduced performance for various reasons, e.g., due to illness or work environment-related factors hindering the individual to perform at full potential. This type of production loss at work is also referred to as presenteeism [36]. Several studies point towards a relationship between health and productivity in the workplace [37,38,39], and working while ill is related to increased risks for coronary events [40], future ill health [41], and future sick absence [42,43]. Production loss due to psychosocial work environment factors such as leadership, social climate, and role clarity has been used in previous studies. Lohela-Karlsson et al. [6], for example, argue that work environment-related production loss can be used to screen organizations regarding production loss that has to do with the work environment. The measure can also function as an outcome measure for interventions aimed at improving organizational work environment. Factors that are linked to the work environment (leadership, social climate, opportunities for influence, requirements, social support, and noise) have a greater impact on production loss than factors that are linked to health [44]. 

In order to investigate the organizational and social work environment, instruments allowing participants to respond with open-ended answers describing their perceived work environment have been used by organizations. Josephson and Vingård [45] investigated the use of such non-validated but frequently used instruments for examining the organizational and social work environment in workplaces. They investigated the use of perceived attitude and perceived influence in such instruments and compared results with the demand control support model [4]. The results show that a high opportunity to influence, combined with a positive attitude, also presupposes lower job strain and greater social support.

In order to evaluate and systematically capture work environment factors in a more actionable way, the STAMINA model was created [46]. The model is a structured support model for the mandatory steps in the systematic work environment management and includes support for gathering information, analyzing risks, creating action plans, and following up on implemented measures. The model constitutes a systematic approach to dealing with different aspects of work environment issues. As part of the model, using results from Josephson and Vingård [45], the human resources index was created (HRI). HRI uses an open-ended question as input, where the employee can choose what characterizes his or her work environment. This makes the question easy to use and reduces the time required by employees to fill out the questionnaire. HRI results in a single measurement of the perceived work environment at a given point in time. The HRI measure is part of a process that aims to increase the employees’ participation, involvement, and understanding of the work environment’s importance for health, efficiency, effectiveness, and well-being [46]. Since the HRI measure is an integrated part of this support model, changes in the measure can be used to evaluate how the work environment has changed after the introduction of various active measures. The organization can then implement the stages of the statutory systematic work environment work: risk assessment, measures, and follow-up [1]. Previous research indicates the importance of employee participation in work environment-related processes [45,47,48]. Processing the results in a participatory process and having an objective measure that is easy to use signifies the core of the STAMINA-process [46]. 

Questions yet to be answered relate to how the measure of HRI correlates with previously used measures of work environment (e.g., relational justice, short-term recovery, and self-reported production loss) and how Swedish municipal employees perceive their organizational and social work environment using the HRI measure. Since HRI is used as a process feedback measurement in a structured support model for systematic work environment management in work groups, it is essential to investigate how the HRI measure correlates with other important work environment related measures. This study is the first study investigating the HRI measure as a process feedback measurement for work environment management and constitutes a first descriptive study of the measure. 

The aim of the study is to estimate the level of the human resources index (HRI) measure among Swedish municipal employees, and to investigate the association between human resources index (HRI) and previously used work environment measures using a sample of employees from eighteen Swedish municipalities.

## 2. Materials and Methods

### 2.1. Study Aim and Hypotheses

The aim of the study is to estimate the level of the human resources index (HRI) measure among Swedish municipal employees, and to investigate the association between human resources index (HRI) and previously used work environment measures (relational justice, short-term recovery, work environment-related production loss, and health-related production), using a sample of employees from eighteen Swedish municipalities. Specifically, two main hypotheses are tested:

**Hypothesis** **1.** *Human Resources Index is positively correlated with (a) perceived relational justice and (b) short-term recovery*.

**Hypothesis** **2.** *Human Resources Index is negatively correlated with (a) work environment-related production loss and (b) health-related production loss*.

The study was conducted within the framework of the STAMINA-project. More information regarding the STAMINA-project is found in the previously published study protocol [46]. 

### 2.2. Study Design and Participants

The study uses a sample from employees in eighteen Swedish municipalities (including both white collar and blue-collar workers). The study is designed as a cross-sectional study of human resources index (HRI) as a dependent variable, and its relationship to several independent variables, i.e., short-term recovery, relational justice, work environment-related production loss, and health-related production loss. 

Municipalities in Sweden were eligible for the study and the selection of municipalities was made as to achieve variation in number of employees and geographical conditions. The study used no specific sampling procedure, instead eligible participants were municipal employees working with the STAMINA-model [46]. (Demographical data for the participating municipalities is found in Appendix A). The participating municipalities had the opportunity to choose the number of employees included in the study. The study sample consisted of work groups applying the model in their systematic work environment management. Therefore, no information regarding the individual employees was processed or analyzed.

Data were collected between January 2017 and March 2018. Ethical approval of the study was given by the Regional Ethical Review Board in Uppsala, Sweden (ref. nr. 2017/093).

### 2.3. Measurements

All survey items were digitally distributed to the participants. Participants were invited to take the online survey by their supervisor via e-mail. The survey consisted of one open-ended item and 11 additional items. (Details of the online survey can be found in Appendix C).

#### 2.3.1. Human Resources Index (HRI)

Human resources index is formed by adding together the answer to the open-ended question: How would you describe your current work situation? Each participant may give up to seven free-text answers to the question. The respondents state their attitude (valence) and their perceived control (opportunity to influence) on an unnumbered continuum for each answer (which implicates a value of 1–100). For each individual answer, an HRI-value was computed. Control carries a higher weight when calculating HRI. The mean HRI score of separate answers for each individual was then calculated creating the human resources index, which can take on a value between 1 and 100. HRI > 50 indicates a good work environment and HRI < 50 indicates a poor work environment. Previous studies have used similar techniques for determining valence for environmental factors [49] and open-ended questions and rating of attitude and influence [45]. 

#### 2.3.2. Categorization of Free-Text Answers

When the respondents answer the HRI question, their free-text responses can be categorized by the respondent into one of ten different categories: (1) Results and goal fulfilment, (2) external circumstances and the outside world, (3) implementation and follow-up, (4) work environment and health, (5) roles and tasks, (6) skills and learning, (7) demands and feedback, (8) time use and working methods, (9) communication and collaboration, and (10) other. The choice of category provides information on which parts of the work environment that are characteristic for the participants’ working day. 

#### 2.3.3. Short-Term Recovery

Short-term recovery (i.e., sleep quality) is measured with one item from the Karolinska sleep questionnaire [50]: Have you experienced any of the following complaints in the past three months? Not feeling refreshed when waking up (KSQ: Question 9 h). The scale is a six-point scale with the options: never, rarely, sometimes, often, mostly, and always. When reporting results from the question, an inverted scale was used, where 1 characterizes poor recovery and 6 characterizes good recovery. The question was used to assess short-term recovery and was used in this study as a marker for recovery and resistance to stress-related ill health. This measure was chosen because recovery is closely linked to the individual experience of a situation and is an interactive process between the individual and the environment [51]. 

#### 2.3.4. Work Environment-Related Production Loss

Perceived productivity is measured using two validated questions that capture the effect of work environment-related problems on performance. Work environment problems were measured with one item [6]: Over the past 7 days, have you experienced any work environment-related problems? Work environment problems refer to all possible physical, psychological, or social problems that may arise in the work environment. The answer option was yes/no. If the respondent answers yes, a follow-up question is posed, where the respondent can estimate the degree of work environment problems:

During the last seven days, how much did your work-environment problem affect your performance while you were working? Think about days where you were limited in the amount or kind of work you could do, days where you accomplished less than you would like, or days where you could not do your work as carefully as usual. If work environment-related problems affected your work very little, then choose a low number. Choose a high number if work environment-related problems affected your work a great deal.

The respondent was asked to estimate the degree of problem on a ten-point scale, where: 1 = had no impact; 10 = prevented me completely. Based on this estimate, it is possible to make a calculation of the extent of the impact of a negative work environment on productivity and performance. This measure was chosen because when intervening for a better working environment, workplace productivity may be affected. It is important to capture the individual’s perception on changes in the work environment and asking about productivity is therefore part of the intervention [46].

#### 2.3.5. Health-Related Production Loss

Perceived productivity is measured using two validated questions that capture the effect of health-related problems on performance. Health problems related to productivity were measured with one item [6]: Over the past 7 days, have you experienced any health-related problems at work? Health problems refer to all possible physical and emotional problems or symptoms. The answer option was yes/no. If the respondent answers yes, a follow-up question is posed, where the respondent can estimate the degree of the health problems:

During the past 7 days, how much did your health problems affect your performance while you were working? Think about days where you were limited in the amount or kind of work you could do, days where you accomplished less than you would have liked, or days where you could not do your work as carefully as usual. If the health problems affected your work very little, then choose a low number. Choose a high number if the health problems affected your work a great deal.

The respondent was asked to estimate the degree of the problem on a ten-point scale, where: 1 = had no impact; 10 = prevented me completely. Based on this estimate, it is possible to make a calculation of the extent of the impact of ill health on productivity and performance. This measure was chosen because when intervening for a better working environment, workplace productivity may be affected. It is important to capture the individual’s perception on changes in the work environment and asking about productivity is therefore part of the intervention [46].

The question is based on the Swedish version of Work Productivity and Activity Impairment—General Health questionnaire (WPAI-GH) [52]. 

#### 2.3.6. Relational Justice Index (RJI)

The scale measuring relational justice has been used by researchers to estimate leadership behaviors that employees perceive as fair and has previously been used to indicate leadership that promotes a good work environment. The index is measured with six items, which are then converted into a relational justice index (RJI). The questions have been developed and validated by Elovainio et al. [53]. Sample item: Please answer the following questions about the general behavior of your supervisor at work. Use the following scale: from 5 = strongly agree to 1 = strongly disagree. 1. Your supervisor considered your viewpoint. A summation index is created, and RJI can vary in the range between 6 and 30. In previous research, the index has also been shown to be closely linked to other psychosocial outcomes (see, for example, (16) [16,20]. Questions about leadership and the attitude of the manager are included in several accepted instruments for measuring the work environment (see, for example, QPS Nordic [54]). Complete survey items for relational justice can be found in Appendix B.

Table 1 provides a summary of used constructs and sample items used in the study.

### 2.4. Data Analysis

Data were analyzed using R-studio [55]. Descriptive statistics for each construct are presented as mean (SD). Significance level was set to *p* < 0.01. Respondents with any missing values were removed. For the relational justice construct (six items), internal consistency was calculated (α = 0.871). Relational justice index was calculated by adding the scores of the six items for each respondent. Parametric assumptions were checked for each variable. For continuous variables, *t*-test and Pearson r correlation were calculated. For categorical variables, Wilcoxon rank sum and Spearman rank were calculated. Effect size was calculated using Cohen’s d.

## 3. Results

### 3.1. Participants

A total of 6402 individuals from 18 different Swedish municipalities participated in the study by completing an online questionnaire. The sample consists of 7004 individuals from eighteen municipalities. When correcting for missing values, 6402 respondents remained in the final data set. Demographical data from the sample can be found in Appendix A. 

### 3.2. Descriptive Statistics

The mean (SD) for each of the variables is reported in Table 2. Mean HRI from the sample was 59.78 (20.31). For relational justice index, Cronbach’s Alpha from the sample were 0.871. Percentage of respondents reporting health-related production loss was 48%, and percentage of respondents reporting work environment-related production loss was 49%. Thus, the prevalence of presenteeism among municipal employees was close to 50% in the sample. The proportion of respondents having an HRI below 50.0 was 28%.

### 3.3. Associations between Variables

Associations between HRI and short-term recovery, relational justice index, work environment-related production loss, and health-related production loss were calculated. HRI is shown to be positively associated with short-term recovery and with relational justice index, thus finding support for hypotheses 1a and 1b. HRI was negatively associated with work environment-related production loss and health-related production loss, thus supporting hypothesis 2a and 2b. The strength of the association was weak for short-term recovery and health-related production loss (r = 0.271 and r = −0.226). The association was moderate for RJI and work environment-related production loss (r = 0.314 and r = −0.366). The results are presented in Table 3.

### 3.4. Effect Size for Self-Reported Health-Related Production Loss

Respondents reporting health-related production loss and work environment related production loss were compared with respondents not reporting health-related production loss and work environment related production loss. Table 4 shows the effect size of not having reported health-related production loss and not having reported work environment related production loss. The effect size for HRI was medium (d = 0.425) for health-related production loss and large (d = 0.712) for work environment related production loss. The effect size was small (d = 0.310 and 0.478 respectively) for RJI. For short-term recovery, the effect size was large (d = 0.858) for health-related production loss and medium (d = 0.498) for work environment related production loss.

### 3.5. Categorization of Free-Text Answers

Respondents were asked how they would like to categorize their answers to the open-ended question. There were 31,097 unique answers in the sample (M = 4.86). Distribution of answers among ten categories is presented in Table 5. Most of the answers (31.5%) were reported in the category of work environment and health. The second and third most reported categories were roles and tasks (13.8%) and communication and collaboration (11.8%). Table 5 provides a presentation of the categorization of free-text answers among the respondents and the proportions of those who reported a positive attitude in the respective categories.

Of the submitted answers, a positive attitude was reported in six of the ten possible categories, and the proportion of submitted answers ranged from 45.2% positive for the category of demands and feedback to 72.1% positive for the category of skills and learning. A negative attitude dominated in four of the categories.

## 4. Discussion

The aim of the study was to estimate the level of the human resources index (HRI) measure among Swedish municipal employees, and to investigate the association between human resources index (HRI) and relational justice, short-term recovery, work environment-related production loss, and health-related production loss.

In this study, the HRI measure in itself was shown to be associated with relational justice, short-term recovery, and perceived productivity and may be used as an indicator that the process creates changes relevant for the employees. The use of the HRI measure in other studies indicates that taking the experiences of the employees as a starting point provides valuable information and input to a constructive discussion about the work environment of a work group [48]. Qualitative results from previous studies on systematic work environment management also show that working together as a work group with these issues creates a shared platform of communication and a collective sense-making of the current work environment, which develops a shared understanding in the work groups for common work environment issues that need to be addressed [48,56,57].

When employees are given an opportunity to evaluate aspects of their work environment with the use of open-ended free-text answers, the results come closer to the experiences of the employees and thus are easier to interpret and decipher. Other studies have identified the mediating effect of psychological safety on engagement and job performance, highlighting the need to develop a climate of psychological safety [58] before addressing issues of resources and other working conditions [59]. Concern for the social environment is identified as an important factor in preventive occupational behavior at work [60]. In this study, this is confirmed by showing the link between relational justice (leadership) and HRI. The social climate of a workplace is highly influenced by the perceived relational justice among the employees [15,25], and first-line managers play an important role in the development of the social work environment at a workplace [56]. The results show that when the employees categorize their free text answers, they focus on the work environment and health (32%), communication and relationships (14%), and roles and tasks (12%). This indicates the importance of social climate and of clarity in roles and tasks and its effect on the perceived overall working environment. Previous studies have indicated the importance of role clarity [61], which is confirmed in the present study and the involvement of employees in work environment endeavors [45,47,48].

An important part of the work according to the STAMINA model is that the employees jointly identify situations and behaviors at their workplace that need to be reviewed from a work environment perspective. These situations may be linked to physical, social, or organizational aspects [46]. Focus for the process is thus on the work group level and on areas that need to be addressed and actively developed in the work environment. Frequently used and popular work environment apps are often focused on the individual level and usually lack clear indications of how to use gathered data and what measures need to be taken [62]. In addition, several health-related apps lack in both clinical evidence [63] and long-term engagement from the users [64]. The structured support model makes it possible to capture important issues that the employees identify as problematic in their work environment and use as an input in a systematic process for developing the work environment [48]. The often-used phrase “what gets measured, gets done” does not apply to work environment issues. It is much easier to measure than to implement the right measures.

Previous studies have argued that quantitative work environment surveys need to be complemented with qualitative investigations [65]. However, since such investigations are costly, in terms of manpower, and time consuming, using an open-ended question such as the open-ended questions underlying the HRI index/measure may prove important when investigating the experienced work environment. The HRI measure is part of a support model used in workplaces to systematically address work environment-related issues [46]. By monitoring changes in the HRI measure, it is possible to determine whether the measures taken effect production loss, perceived leadership, and short-term recovery in a work group. The support model using HRI may thus function as a complement to biannual work environment surveys since it also, as shown in the present study, captures important aspects of the work environment such as productivity, relational justice, and short-term recovery. The support model using HRI may thus be used to capture and investigate the overall well-being and possibly even performance using a measure that is easy to compile and easy to interpret. Open-ended answers can be used as an engine to maintain an ongoing work environment process and the HRI measure can thus be used to approach work environment development in work groups. Having a single measure that is simple to follow, may help to create commitment and engagement for work environment issues among participating employees.

The results from this study suggest that the HRI measure is a useful process feedback measurement that can be used to address several issues related to occupational health. We believe that the results support the notion that HRI can bring interesting insights and be a useful measure when intervening in work groups using a structured support model for systematic work environment management.

### Limitations

The study group demographics show a bias towards female municipal employees. Further research in other organizations, both public and private sector, is needed to understand and validate the results. Generalization is thus not possible beyond the traditional municipal responsibilities: healthcare, day-care, education, and municipal administration. However, the results from this study give a good indication of the human resources index among Swedish municipal employees.

Although this is not a validation study, we argue that the HRI scale is a useful tool to address issues related to occupational health. In order to strengthen this tentative conclusion, further evaluation of the instrument might be necessary. For example, the lack of sociodemographic data means that results from the present study may need to be further elaborated using a design allowing for validation of the measurements with the possibility to control for sociodemographic data.

Eligible participants were employees taking part in the STAMINA-project and were not randomly selected. This is a limitation of the study implying that the results may look different had a random sampling procedure been used. In addition, the study had a cross-sectional design, which has some disadvantages. Since data was gathered at one point in time made it difficult to draw casual inference, and it was difficult to investigate the temporal relation between variables [66].

## 5. Conclusions

The findings implicate that HRI captured important aspects of the work environment such as productivity, relational justice, and short-term recovery. The HRI measure is part of a structured support model used in workplaces to systematically address work environment-related issues. Monitoring changes in the HIR measure makes it possible to determine whether the measures taken affect production loss, perceived leadership, and short-term recovery in a work group. The support model using HRI may thus be used to complement traditional work environment surveys conducted in Swedish organizations as obliged by legal provisions.

## Figures and Tables

**Table 1:** Sample survey items.

Construct	Sample Item	Previous Source(s)
HRI Human Resources Index	How would you describe your current work situation?	N.A.
Short-term recovery	Have you experienced any of the following complaints in the past three months? Not feeling refreshed when waking up.	Karolinska Sleep Questionnaire (KSQ) [50] Åkerstedt et al. (2008)
Relational Justice Index	Your supervisor considered your viewpoint.	[53] Elovainio et al. (2002)
Work environment-related production loss	During the last seven days, how much did your work-environment problem affect your performance while you were working?	[6] Lohela-Karlsson et al. (2013)
Health-related production loss	During the last seven days, how much did your health problem affect your performance while you were working?	[6] Lohela-Karlsson et al. (2013)

**Table 2:** Mean (SD) results for human resources index, relational justice index, short-term recovery, work environment-related production loss, and health-related production loss.

Variable	Scale	(*n* = 3121) Mean	SD
Human Resources Index (HRI)	1–100	59.78	20.31
Relational Justice Index *	6–30	24.96	4.18
Short-term recovery *	1–6	3.71	1.20
Production loss (health)	1–10	3.05	2.43
Production loss (work environ.)	1–10	3.41	2.45

* Inverted items: high value indicates good recovery and high relational justice.

**Table 3:** Association between HRI and short-term recovery, RJI, work environment related production loss, and health related production loss.

	Variable(s)	Short-Term Recovery ^‡^	RJI ^†^	Production Loss (Work Environ.) ^‡^	Production Loss (Health) ^‡^
(*n* = 3121)	Human Resources Index (HRI)	0.271 *	0.314 *	−0.366 *	−0.226 *

^‡^ Spearman rank, ^†^ Pearson’s r, * *p* < 0.001.

**Table 4:** Effect size for self-reported health-related production loss and HRI, RJI, and short-term recovery.

**Variable**	**Did Report Health-Related Production Loss** **(*n* = 3077)** **Mean (SD)**	**Did Not Report Health-Related Production Loss** **(*n* = 3325)** **Mean (SD)**	**Test of Difference** (*t*-Test) *	**Effect Size** **(Cohen’s d)**
HRI	55.40 (20.47)	63.84 (19.29)	T = 19.98	0.425
RJI	24.29 (4.44)	25.57 (3.83)	T = 12.38	0.310
Short-term recovery	3.20 (1.15)	4.18 (1.06)	T = 35.48	0.858
	**Did report Work Environment RELATED Production Loss** **(*n* = 3146)**	**Did Not Report Work Environment Related Production Loss** **(*n* = 3256)**		
HRI	52.85 (20.0)	66.48 (18.25)	T = 28.50	0.712
RJI	23.95 (4.47)	25.93 (3.63)	T = 19.49	0.487
Short-term recovery	3.42 (1.18)	4.00 (1.15)	T = 19.92	0.498

Note: Health-related production loss: Have you experienced health problems at work? (yes/no) Work environment related production loss: Have you experienced work environment related problems? (yes/no). * *p* < 0.001.

**Table 5:** Respondents’ categorization of free-text answers.

Category	(*n* = 31,097) %	Proportion of Positive Attitude %
(1) Results and goal fulfilment	7.0	65.5
(2) External circumstances and the outside world	6.0	45.7
(3) Implementation and follow-up	6.7	67.0
(4) Work environment and health	31.5	47.0
(5) Roles and tasks	13.8	69.6
(6) Skills and learning	9.9	72.1
(7) Demands and feedback	3.1	45.2
(8) Time use and working methods	7.3	48.3
(9) Communication and collaboration	11.8	69.5
(10) Other	2.9	53.5

Note: A proportion >50.0% indicates a majority of positive attitude. A proportion < 50.0% indicates a majority of negative attitude.

## Data Availability

The data presented in this study are available in Supplementary Material.

## Supplementary Materials

The following are available online at https://www.mdpi.com/article/10.3390/ijerph18126509/s1, sample_data.xls.

## Appendix A

**Table A1:** Number of citizens and number of municipal employees in participating municipalities.

Information About Municipalities	Participants (*n* = 18) Mean (Range)
Number of citizens	96,977 (8997–347,949)
Number of employees	6581 (1002–23,941)

**Table A2:** Number of employees and age structure among the employees in participating municipalities.

Municipality	No. of Employees (2019)	Women (%)	Men (%)	<35 y (%)	35–54 y (%)	>54 y (%)	Employees with Higher Diploma (%)
A	2136	78	22	25	44	31	39
B	6519	79	21	25	45	27	54
C	3302	77	23	23	46	30	45
D	2804	79	21	27	48	26	58
E	6660	75	25	27	48	25	60
F	2432	70	30	25	47	28	60
G	9070	77	23	28	47	25	61
H	23,941	74	26	31	46	23	60
I	4290	77	23	28	46	26	57
J	10,126	80	20	29	47	24	52
K	3299	79	21	27	46	27	50
L	7285	74	26	28	47	25	47
M	4469	78	22	31	46	23	47
N	2376	77	23	24	51	25	54
O	9292	70	30	31	46	23	60
P	1002	72	28	31	45	24	48
Q	8159	78	22	25	49	25	57
R	11,300	75	25	31	45	24	54

## Appendix B

Relational Justice

Please answer the following questions about the general behavior of your supervisor at work. Use the following scale: from 5 = strongly agree to 1 = strongly disagree.

1. Your supervisor considered your viewpoint.
2. Your supervisor was able to suppress personal biases.
3. Your supervisor provided you with timely feedback about the decisions and their implications.
4. Your supervisor treated you with kindness and consideration.
5. Your supervisor showed concern for your rights as an employee.
6. Your supervisor took steps to deal with you in a truthful manner.

## Appendix C

Human Resources Index HRI

Step 1. Introduction

The STAMINA model’s web question examines what you consider relevant to describe your current work situation, without using guiding questions or questions that someone else has come up with. Instead, you respond to an open-ended question that reads: “What characterises your work situation right now?” You answer by writing down what you think of until you get a bullet list:

1. Your first answer—a thought or perception;
2. Your second answer;
3. Your third answer;
4. Your fourth answer;
5. Etc.

When you are finished, the bullet list is saved, and each bullet reappears on the screen. Now mark what you think of each point. Does it stand for something you experience as positive or negative and how much do you think you can influence what you have written? You mark your setting using the stepless scale. Each answer should be linked to an area that you select from a drop-down list before proceeding to the next item on your list. Your and your colleagues’ answers are compiled in a report. In the report, you can read all the answers from the group, but no one can see who wrote what. Before you finish, you will be asked to answer a few short questions. They are part of a large research project on how the work environment affects our health and productivity. A research group at Uppsala University follows different workplaces in a large study. You will not be able to see your answers in the group reports, as they are only intended for research.

Step 2. How would you describe your current work situation?

Make a list: Give your personal picture of your work. Describe your experiences, both positive and negative. Make a note of what you will think of in the fields to the right. Use one field for each point. You have seven fields at your disposal. Try to use at least three of the fields. Answer as spontaneously and sincerely as possible. Remember that your answers are anonymous and confidential.

Step 3. Evaluation

We now ask you to evaluate what you have written by clicking or dragging the selection on the scale.

1. You wrote: [STRESS]
What attitude do you have to this that you wrote?
Negative—Positive.
To what extent can you influence what you have written?
Not at all—Completely.
How important is this for you?
Not at all important—Very important.

In which of the following areas/categories do you think what you have written belongs?

1. Results and goal fulfilment;
2. External circumstances and the outside world;
3. Implementation and follow-up;
4. Work environment and health;
5. Roles and tasks;
6. Skills and learning;
7. Demands and feedback;
8. Time use and working methods;
9. Communication and collaboration;
10. Other.

Step 4. Research questions

The STAMINA model contains research questions that Uppsala University poses in order to develop Swedish working life. The answers are compiled in a national database that is part of a multiyear national study. Only the researchers get access to the collected material. Your and your group’s answers will therefore not be reported and are thus not included in the group report that you and your group will have access to. The research questions begin with questions about how you view life in general. Then follows questions about your health and sleep and ends with questions about your relationship with your boss.
